# Detection of Motor Changes in Huntington's Disease Using Dynamic Causal Modeling

**DOI:** 10.3389/fnhum.2015.00634

**Published:** 2015-11-25

**Authors:** Lora Minkova, Elisa Scheller, Jessica Peter, Ahmed Abdulkadir, Christoph P. Kaller, Raymund A. Roos, Alexandra Durr, Blair R. Leavitt, Sarah J. Tabrizi, Stefan Klöppel, TrackOn-HD Investigators, A. Coleman

**Affiliations:** ^1^Department of Psychiatry and Psychotherapy, University Medical Center FreiburgFreiburg, Germany; ^2^Freiburg Brain Imaging Center, University Medical Center FreiburgFreiburg, Germany; ^3^Laboratory for Biological and Personality Psychology, Department of Psychology, University of FreiburgFreiburg, Germany; ^4^Department of Computer Science, University of FreiburgFreiburg, Germany; ^5^Department of Neurology, University Medical Center FreiburgFreiburg, Germany; ^6^BrainLinks-BrainTools Cluster of Excellence, University of FreiburgFreiburg, Germany; ^7^Department of Neurology, Leiden University Medical CentreLeiden, Netherlands; ^8^Department of Genetics and Cytogenetics, Pitié-Salpêtrière University HospitalParis, France; ^9^Centre for Molecular Medicine and Therapeutics, Department of Medical Genetics, University of British ColumbiaVancouver, Canada; ^10^Department of Neurodegenerative Disease, Institute of Neurology, University College LondonLondon, UK

**Keywords:** Huntington's disease, motor network, sequential finger tapping, fMRI, DCM, cluster analysis

## Abstract

Deficits in motor functioning are one of the hallmarks of Huntington's disease (HD), a genetically caused neurodegenerative disorder. We applied functional magnetic resonance imaging (fMRI) and dynamic causal modeling (DCM) to assess changes that occur with disease progression in the neural circuitry of key areas associated with executive and cognitive aspects of motor control. Seventy-seven healthy controls, 62 pre-symptomatic HD gene carriers (preHD), and 16 patients with manifest HD symptoms (earlyHD) performed a motor finger-tapping fMRI task with systematically varying speed and complexity. DCM was used to assess the causal interactions among seven pre-defined regions of interest, comprising primary motor cortex, supplementary motor area (SMA), dorsal premotor cortex, and superior parietal cortex. To capture heterogeneity among HD gene carriers, DCM parameters were entered into a hierarchical cluster analysis using Ward's method and squared Euclidian distance as a measure of similarity. After applying Bonferroni correction for the number of tests, DCM analysis revealed a group difference that was not present in the conventional fMRI analysis. We found an inhibitory effect of complexity on the connection from parietal to premotor areas in preHD, which became excitatory in earlyHD and correlated with putamen atrophy. While speed of finger movements did not modulate the connection from caudal to pre-SMA in controls and preHD, this connection became strongly negative in earlyHD. This second effect did not survive correction for multiple comparisons. Hierarchical clustering separated the gene mutation carriers into three clusters that also differed significantly between these two connections and thereby confirmed their relevance. DCM proved useful in identifying group differences that would have remained undetected by standard analyses and may aid in the investigation of between-subject heterogeneity.

## Introduction

Huntington's disease (HD) is a genetic neurodegenerative disorder characterized by a devastating combination of motor, cognitive, and psychiatric symptoms, with a typical clinical onset around the age of 40. Advances in genetic testing have offered the opportunity to reliably diagnose the fully penetrant genetic mutation many years before the onset of first symptoms.

A substantial body of research, including large-scale multimodal and multicenter studies, such as PADDINGTON (Hobbs et al., 2013), PREDICT-HD (Biglan et al., 2013), and TRACK-HD (Tabrizi et al., 2009), have revealed a complex pattern of structural and functional abnormalities in diverse subcortical and cortical regions in both pre-clinical (preHD) and early manifest (earlyHD) gene mutation carriers. HD disease-specific effects have been observed in fronto-striatal and fronto-parietal networks (Klöppel et al., 2008, 2010; Rosas et al., 2008; Wolf et al., 2008, 2011, 2012; Tabrizi et al., 2009), affecting essential cognitive, motor and executive domains. Specifically, deficits in motor functioning are a clinical hallmark of HD, as indicated by previous functional magnetic resonance imaging (fMRI) studies (Biglan et al., 2009; Klöppel et al., 2009), and are possibly caused by striatal atrophy as well as volume loss in prefrontal areas (Lawrence, 1998; Rosas et al., 2003). Furthermore, diffusion tensor imaging (DTI) studies have indicated disease-specific changes in overall white matter diffusivity, correlated with caudate and white matter volume loss (Novak et al., 2014), as well as alterations in striatal projection pathways and their associations with clinical motor data (Poudel et al., 2014) in earlyHD and to varying extent in preHD. Moreover, neuronal loss progressively affecting frontal, sensorimotor, and parietal regions appears to be remarkably variable both within and between HD gene carrier sub-populations (Nana et al., 2014).

Despite structural changes, behavioral performance during motor tasks remains relatively intact in individuals far from clinical onset, possibly as the result of compensatory mechanisms, but starts to deteriorate at early stages of manifest HD and during more demanding motor tasks (Farrow et al., 2006; Feigin et al., 2006; Georgiou-Karistianis et al., 2014). Functional MRI has proven to be a promising candidate for studying functional decline as well as neural compensatory reorganization in both preHD and earlyHD. Previous neuroimaging data, including PET studies, have identified HD disease-specific abnormalities in key brain areas involved in motor control, such as the primary motor cortex, supplementary motor area (SMA), premotor cortex, and parietal regions (Bartenstein et al., 1997; Gavazzi et al., 2007; Klöppel et al., 2009). However, findings vary across studies, suggesting that the nature of changes in brain activations is still not well understood. Furthermore, studying distinct, spatially segregated brain areas in isolation may not necessarily provide insights into the inter-regional interactions within functional networks and how connectivity becomes abnormal in clinical conditions in general and specifically in HD.

Functional integration, as opposed to functional segregation, allows us to focus on the dynamic causal interactions between distinct brain regions (i.e., effective connectivity) and how they depend on the task that the brain is performing. One of the most widely used methods for assessing effective connectivity is dynamic causal modeling (DCM) (Friston et al., 2003). DCM is a hypothesis-driven Bayesian approach that has been successfully used to study causal interactions between regions sub-serving the same functional network, as well as the way experimental manipulation influences connectivity in both healthy individuals and clinical populations (for a review of DCM studies in patients see Seghier et al., 2010). In a previous DCM study in preHD (Scheller et al., 2013), we identified an excitatory effect from bilateral dorsal premotor cortex (PMd) to parietal regions as critical for compensation, an effect that was restricted to conditions with high cognitive demand and was most pronounced in individuals closer to clinical onset of first motor symptoms. To our knowledge, this is the only task-based DCM study in HD published in the literature, so far.

Here, we collected motor task fMRI data from 155 participants from the large-scale, multi-centric TrackOn-HD study (Klöppel et al., 2015; http://hdresearch.ucl.ac.uk/completed-studies/trackon-hd/). We used DCM, based on task fMRI, to assess abnormal effective connectivity of the motor network in HD. The aim of the current study was twofold: first, we sought to extend on our previous DCM findings using a much larger and clinically heterogeneous sample. Specifically, previous results (Scheller et al., 2013) indicated the crucial role of the dorsal premotor cortex for the maintenance of motor functioning in preHD. Furthermore, research has shown that impairment in the striatum and its frontal motor projection areas in manifest HD, including the premotor cortex, may induce a compensatory recruitment of parietal cortices (Bartenstein et al., 1997). A differential involvement of the SMA has also been reported (Klöppel et al., 2009), expressed by the over-recruitment of caudal SMA during faster finger-tapping movements with approaching clinical onset in preHD, possibly indicative of its compensatory role, as well as a monotonic attenuation in task-related activity in pre-SMA during complex finger-tapping movements, most likely indicating disease-specific changes. Thus, we here expected to provide further evidence for the compensatory role of premotor and parietal areas, associated with approaching clinical onset and increasing cognitive demand.

Second, and more importantly, we aimed to demonstrate the use of an exploratory cluster analysis based on DCM parameters as a classification method in identifying sub-groups among the HD gene mutation carriers that may benefit from targeted interventions. Furthermore, we investigated to what extent the DCM parameters differed among the identified sub-groups and how differential neural coupling strengths were associated with behavioral performance during the finger-tapping task and clinical markers of disease progression. We hypothesized that effective connectivity would not be homogeneously altered across the group of HD gene carriers but may depend on the task demand and the disease progression in some individuals more than in others.

## Materials and methods

### Study population

A total of 241 participants were recruited within the large-scale, multimodal TrackOn-HD study at four different sites (Paris, London, Vancouver, and Leiden). Out of them, only 155 right-handers completed a sequential finger-tapping motor task. In addition to left-handedness (*n* = 16), further exclusion criteria included technical issues (*n* = 11), corrupt or missing fMRI data (*n* = 9), poor task performance and missing activations (*n* = 15), as well as failed DCM quality check (*n* = 35). A detailed summary of excluded participants is provided in the Table S1.

For the current study, data were available for a total of 155 participants scanned between April and November 2013, comprising the following three groups: 77 age- and gender-matched controls (HC: 45 females, mean age ± SD: 48.53 ± 9.56), 62 individuals without HD but carrying the mutant huntingtin (HTT) gene (preHD: 30 females, mean age ± SD: 41.89 ± 8.58), and 16 early manifest HD patients (earlyHD: 6 females, mean age ± SD: 46.18 ± 8.59). PreHD required a disease burden of pathology score greater than 250 and a total of total motor score of 5 or less in the motor assessment of the Unified Huntington's Disease Rating Scale (UHDRS 99), indicating no substantial motor signs. EarlyHD were required to have motor symptoms consistent with HD, and a diagnostic confidence score of 4, according to the UHDRS, as well as to be within the Shoulson and Fahn stage I or II (Shoulson and Fahn, 1979) assessed according to UHDRS total functional capacity (TFC ≥ 7) (Tabrizi et al., 2009). Demographic and clinical information is provided in Table 1. Putamen volume (adjusted for total intracranial volume), disease burden score (DBS; Penney et al., 1997), and cumulative probability of clinical onset (CPO; Langbehn et al., 2004) were used as markers of HD disease progression.

**Table 1:** **Demographic and clinical information**.

	**HC (*n* = 77)**	**preHD (*n* = 62)**	**earlyHD (*n* = 16)**
Age (years)	48.53 ± 9.56 (27:67)	41.89 ± 8.58 (24:61)	46.18 ± 8.59 (34:67)
Gender (F/M)	45/32	30/32	6/10
CAG length	–	43.19 ± 2.55 (39:50)	43.25 ± 1.73 (41:48)
CPO	–	0.22 ± 0.15 (0.02:0.62)	0.41 ± 0.21 (0.03:0.83)
Disease burden score^*^	–	304 ± 58 (182:457)	347 ± 48 (224:429)
Putamen (TIV-adjusted)	0.58 ± 0.07 (0.40:0.75)	0.50 ± 0.08 (0.29:0.75)	0.42 ± 0.12 (0.24:0.66)

* *DBS = age × (CAG length-35.5) (Penney et al., 1997). Values are given in means ± SD (range), where applicable. HC, healthy controls; preHD, pre-symptomatic HD; earlyHD, early manifest HD; F, female; M, male; CAG, trinucleotide; CPO, cumulative probability of clinical onset; TIV, total intracranial volume*.

The study was approved by the Ethics Committees of the Institute of Neurology, UCL (London), the University of British Columbia (Vancouver), Pierre and Marie Curie University (Paris), and the University of Leiden (Leiden). All participants gave a written informed consent according to the Declaration of Helsinki (World Medical Association, 2013).

### fMRI paradigm

The experimental design of the motor task fMRI was adopted from a previous study (Klöppel et al., 2009) and consisted of a sequential finger-tapping task probing for both executive (movement speed) and cognitive (movement complexity) aspects of motor control (Figure S1). The successful reproducibility of the finger-tapping paradigm across scanning sites has been shown elsewhere (Gountouna et al., 2010).

The task involved metronome-paced sequential finger tapping with their right dominant hand, using the (1) index, (2) middle, (3) ring, and (4) small fingers. Tapping sequences were either simple (i.e., 1-2-3-4) or complex (i.e., 4-2-3-1). With respect to speed, each sequence was paced by metronome clicks presented to the participant via headphones at a rate of either 0.5 or 1.5 Hz, resulting in slow or fast sequences, respectively. In addition to the task condition, a rest condition was used in which the metronome clicks were presented to the participants but no movement was required. Thus, the experimental paradigm consisted of six types of different blocks, each lasting for 20 s (i.e., simple-slow, simple-fast, complex-slow, complex-fast, rest-slow, and rest-fast). Each block type was presented five times in a pseudo-randomized order.

Button presses during the task were recorded using Current Designs button boxes (http://www.curdes.com). Similarly to our previous study (Klöppel et al., 2009), single omitted or wrongly added button presses were counted as one mistake. In sections with more complex errors, only sequences of three or more buttons in the appropriate order were counted as correct. Participants who scored low in performance (<50% accuracy across all blocks) or performed a completely wrong condition (e.g., simple instead of complex sequence or pressed during the whole rest condition) were excluded from subsequent analyses. It was furthermore examined whether exclusions were dictated by group affiliation using Pearson's chi-square test in SPSS.

Performance from the tapping conditions, measured by the mean timing inaccuracies (i.e., cue-response intervals) and their standard deviations (SD) were compared among the three groups. The timing inaccuracies were calculated from the intervals between each sound click (i.e., cue) and the actual button click (i.e., response) for each participant. Statistical analysis was conducted in SPSS using a 3 × 2 × 2 repeated measures analysis of covariance (ANCOVA), with group (HC, preHD, and earlyHD) as a between-subject factor and complexity (simple and complex) as well as speed (slow and fast) as within-subject factors, adjusting for age, gender, education, and site. Additionally, we investigated the association between performance and CPO among the gene carriers using Pearson's partial correlation, correcting for the covariates.

Mean timing inaccuracies and their SD were chosen as indices for motor performance based on previous literature (Hinton et al., 2007; Klöppel et al., 2009), which showed that motor timing variability, but not accuracy, increased in preHD with approaching clinical onset. Timing inaccuracies, rather than reaction time, reflect the ability of patients to anticipate the next click. However, for reasons of completeness, between-group differences were also investigated for accuracies (percentage of correct responses) for each condition using non-parametric Kruskal-Wallis H test in SPSS.

### MRI data acquisition and preprocessing

Scanning was performed on a 3T Siemens MAGNETOM TimTrio MR scanner at Paris and London and on a 3T Philips Achieva MR scanner at Vancouver and Leiden, both using a 12-channel head coil. High-resolution three-dimensional T1-weighted structural scans were acquired for all participants with a magnetization-prepared rapid gradient echo (3D MPRAGE) sequence for Siemens and a fast-field echo (FFE) sequence for Philips, using standardized protocols with the following parameters for the two scanner systems, respectively (Siemens / Philips): TR = 2200/7.7 ms, TE = 2.9/3.5 ms, TI = 900/875 ms, FA = 10/8°, FOV = 28/24 cm, matrix size of 256 × 256 × 208/224 × 224 × 164, zero-filled in the 3rd dimension to give an isotropic resolution of 1.1 mm. Two T1-weighted scans were acquired for each participant if time allowed and the one with the best quality was used for the analysis. Image quality of the anatomical scans was ensured after visual inspection. For the fMRI motor task, 225 whole-brain volumes were acquired using a T2^*^-weighted single-shot gradient echo planar imaging (GE-EPI) sequence with the following parameters: TR = 3 s, TE = 30 ms, FOV = 212 mm, flip angle = 80°, 48 slices in ascending order (slice thickness: 2.8 mm, gap: 1.5 mm, in plane resolution 3.3 × 3.3 mm), matrix size of 64 × 64, and bandwidth of 1906 Hz/Px. Rigorous inspection of the functional image quality was conducted using the FBIRN QC protocol (Greve et al., 2011; Glover et al., 2012). FBIRN's standardized ratings were based on two summary variables: (1) the number of volumes with mean intensity more than 3 SD away from intensity of overall mean image, and (2) number of volumes with mean volume difference (volume minus overall mean image) of more than 1%. Datasets with more than 20% outlier volumes in at least one variable were excluded from subsequent analyses.

Data preprocessing was performed in SPM8 (Statistical Parametric Mapping, r5638, Welcome Trust Centre for Neuroimaging, http://www.fil.ion.ucl.ac.uk/spm), using MATLAB R2012a (Mathworks, Natick, MA, USA). Each participant's T1 scan was segmented into gray and white matter using the VBM8 (r435) toolbox (http://dbm.neuro.uni-jena.de/vbm/). Segmented images were used to create an improved anatomical scan for co-registration of the functional scans. Using the DARTEL extension (Ashburner, 2007) for high-dimensional registration within the VBM8 toolbox, deformation parameters were extracted for later normalization of contrast images prior to second-level analysis.

The first four functional volumes were discarded prior to data preprocessing to allow for the equilibration of T1 signal effects. The remaining images were realigned to the mean image using a rigid body transformation and co-registered to the improved anatomical scan. Volumes with significant artifacts were detected using the ArtRepair software (http://cibsr.stanford.edu/tools/human-brain-project/artrepair-software.html). Those scans with more than 1.3% variation in global intensity and 1.0 mm/TR scan-to-scan motion were identified as outliers and replaced by interpolation from the nearest unaffected volumes. On average, <3% of all slices for all participants were corrected by this procedure. Following a histogram-based approach for outlier identification, participants with more than 13% of bad volumes were excluded from the subsequent analysis. The co-registered and repaired functional scans were then spatially smoothed with an isotropic Gaussian kernel of 6 mm FWHM.

### GLM analysis

Statistical analysis at the first (within-subject) level was carried out using the General Linear Model (GLM) as implemented in SPM8 (Friston et al., 1994). Task-related changes of BOLD signals were estimated at each voxel by modeling each block separately for each of the conditions (simple-slow, simple-fast, complex-slow, complex-fast, rest-slow, and rest-fast) after convolving with the canonical hemodynamic response function (HRF). High-pass filter with a cut-off at 152 s was applied to the data to remove low frequency artifacts. The instruction screen and the blocks during which participants performed a wrong condition (i.e., accuracy was below 50%) were modeled as separate regressors of no interest. Similarly, single button presses during the rest conditions were modeled as separate regressors. Six additional regressors containing the absolute values of the first derivative of the respective realignment parameters (Power et al., 2012) were included to regress out variance caused by translational and rotational head movements in x-, y-, and z-direction.

Subject-specific contrasts of interest were created from the beta estimates coding the effect of complexity (complex vs. simple sequence), as well as the effect of speed (fast vs. slow sequence). These contrasts were normalized to standard Montreal Neurological Institute (MNI) space using the DARTEL deformation parameters and taken forward to random-effects group analyses, treating participants as a random variable. To reduce inter-subject variability and allow for group analyses, the contrasts were additionally smoothed, resulting in total spatial smoothing of 8 mm FWHM.

Main effects of experimental task were characterized in SPM8 using one-sample *t*-tests, separately for complexity and speed, including age, gender, education, and site as confounding covariates. All participants were included in the one-sample *t*-tests as one group to ensure that regions of interests (ROIs) for the subsequent DCM analysis were commonly activated across all groups. Task-specific activations were identified at *p* < 0.05 FWE-corrected. Additionally, between-group comparisons were implemented in the GLM Flex tool (http://mrtools.mgh.harvard.edu/index.php/GLM_Flex) using a 3 × 2 × 2 ANCOVA design, including group (HC, preHD, and HD) as a between-group factor, as well as complexity (complex and simple) and speed (fast and slow) as within-group factors, while correcting for age, gender, site, and education. In contrast to classical SPM8 analysis, which has a pooled error term across all within-subject factors, GLM Flex uses partitioned error terms and can be used to run full-factorial models with more factors than SPM8 allows.

### DCM analysis

Effective connectivity analysis was conducted using DCM (Friston et al., 2003), a hypothesis-driven Bayesian approach that describes the biophysical nature of directed interactions among distinct brain regions by incorporating two forward models: one at the neural and one at the hemodynamic level. At the neural level, DCM is expressed by the following equation:

$$\begin{array}{l} \frac{dz}{dt}=\left(A+\sum u_{j}B^{j}\right)z+Cu \end{array}$$

where vector *z* represents the time series of the neural behavior, vector *u* contains the time course(s) (*1, …,j, …, n*) of the external perturbation (i.e., the experimental paradigm), as well as the task-independent endogenous couplings denoted by *A*, modulatory effects on these connections by stimulus *u*_*j*_ given by *B*, and experimental input to the system that drives regional activity, modeled by *C*. The hemodynamic model, on the other hand, is based on a biophysical forward model (Balloon model; Buxton et al., 1998) and comprises parameters characterizing blood flow and oxygenation change, measured by the actual BOLD response. By combining a priori knowledge of a biologically plausible neural model (input) with the measured BOLD response (output), it is possible to infer on underlying hidden states such as regional causal interactions. Further reading on the DCM approach can be found elsewhere (e.g., Penny et al., 2004; Friston, 2009; Stephan et al., 2010; Daunizeau et al., 2011; Kahan and Foltynie, 2013).

Here, we used deterministic, bilinear, one-state DCM to assess the effective connectivity among seven regions activated by the motor task (see Table 2 for results) and in agreement with previously published data (Klöppel et al., 2009; Scheller et al., 2013). These regions comprised the left motor cortex (lM1), SMA, divided in pre- (pSMA) and caudal (cSMA), as well as bilateral dorsal premotor cortex (lPMd, rPMd), and bilateral superior parietal cortex (lSPC, rSPC). For each participant, time series from each of the seven ROIs were extracted using the fixed coordinates from the second-level activations identified in the one-sample *t*-test and adjusted for the effect of interest (F-contrast). No statistical threshold was used within each ROI, which allowed for the time series extraction of the same set of voxels in all participants. The motivation for this approach is based on previous literature (Parker Jones et al., 2013) and is advantageous for the current study because it ensured that there was no overlap of subject-specific spheres in neighboring brain regions, which would have otherwise been problematic in the case of pre- and caudal SMA. Furthermore, participants having ROIs with weak activations do not have to be excluded but at the expense of potentially including condition-independent noise (Parker Jones et al., 2013). This is an issue particularly in small sample sizes but potentially less so in our relatively large study.

**Table 2:** **Imaging results: task-specific regions of interest**.

**Regions**	**Hemi-**	**MNI coords**			**T**	**p_*FWE*−*corr*_**
	**sphere**	**(mm)**				
		**x**	**y**	**z**		
Pre-supplementary motor area (pSMA)	L	−8	11	45	12.10	<0.001
Caudal supplementary motor area (cSMA)	L	−5	−5	51	15.54	<0.001
Primary motor cortex (lM1)	L	−38	−12	53	16.46	<0.001
Dorsal premotor cortex (lPMd)	L	−24	−4	46	15.07	<0.001
Dorsal premotor cortex (rPMd)	R	26	−3	47	13.76	<0.001
Superior parietal cortex (lSPC)	L	−16	−63	58	12.93	<0.001
Superior parietal cortex (rSPC)	R	15	−66	58	16.65	<0.001

**Figure 1:**
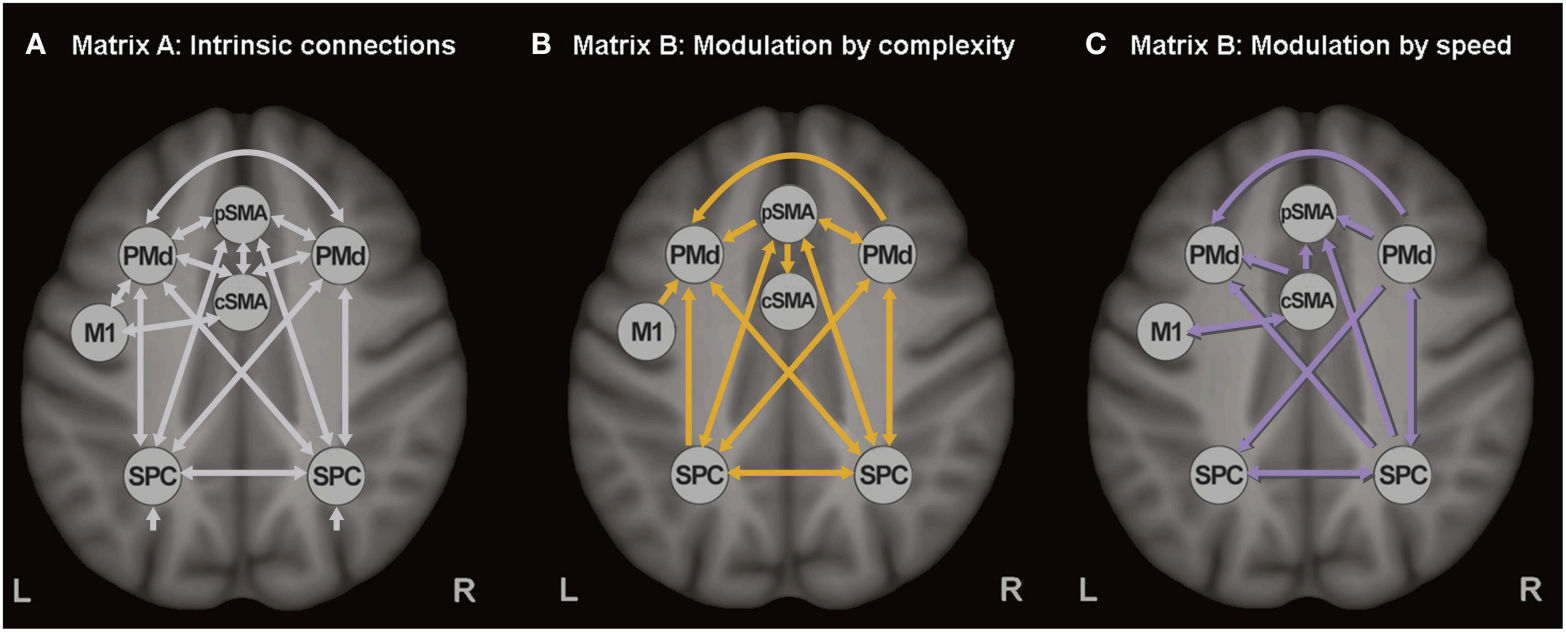
**Dynamic causal model. (A)** Task-independent, intrinsic connections, **(B)** Modulatory connections (complexity), and **(C)** Modulatory connections (speed).

The extracted time series of all seven ROIs were included in one DCM, based on our previous study (Scheller et al., 2013). Intrinsic connections were modeled among all seven regions (represented with white arrows in Figure 1A). SMA was divided in pre-SMA and caudal SMA, the former involved in more cognitively challenging conditions (thus more strongly activated by the complex finger tapping condition) and interconnected with the premotor and associative cortices, while the latter strongly interconnected with M1 and activated by the speed conditions (thus representing the motoric executive part of SMA). No direct connection was modeled between pre-SMA and M1, but an indirect influence was assumed via left PMd and M1. Modulatory connections were specified in the B-matrix, separately for the complex (Figure 1B) and the speed conditions (Figure 1C). Based on the previously reported activations (Klöppel et al., 2009), modulatory effects of speed were included for cSMA, left M1, right PMd, and right SPC, while modulation by complexity was specified for M1, pSMA, right PMd, and bilateral SPC. Modulatory effects were expected only for the right PMd because of its involvement during auditorially paced finger-tapping sequences (Witt et al., 2008), as well as its higher recruitment during more demanding tasks (Bartenstein et al., 1997; Klöppel et al., 2009). All experimental inputs entered the model via the associative sensory regions in the parietal cortex. A more detailed discussion about the choice of connections can be found elsewhere (Scheller et al., 2013).

The fully connected DCMs were then reduced using the *post-hoc* optimization procedure for approximating model evidence, proposed by Friston and Penny (2011). This approach optimizes only the large model, while the evidence for any sub-model is obtained using generalization of the Savage-Dickey density ratio (Dickey, 1971; for more detailed discussion readers may refer to Rosa et al., 2012, Friston and Penny, 2011, and Seghier and Friston, 2013). Additionally, *post-hoc* diagnostics of each participant's DCM were conducted using in-house MATLAB routines to ensure that model inversion has converged, requiring at least 10% of variance explained.

DCM model specification, estimation and *post-hoc* optimization were carried out with DCM12, as implemented in SPM12b. Statistical inference on model parameters was conducted in SPSS, Version 20.0 (IBM Corp., 2011). Random-effects inference at the connection level was assessed using ANCOVA analysis after covariate adjustment. Between-group differences were considered significant at a threshold of *p* < 0.001 after accounting for the number of connections (i.e., 30 intrinsic and 17 modulatory). Two-sample *t*-tests were used for *post-hoc* analyses of significant between-group differences, with applying Bonferroni correction for the three groups.

### Cluster analysis

To identify sub-groups differing in connectivity pattern, DCM intrinsic and modulatory parameters across all HD gene mutation carriers were entered into a hierarchical agglomerative cluster analysis, as implemented in SPSS (Burns, 2009). Ward's clustering linkage method (Ward, 1963) was performed on all parameters with squared Euclidean distance as a measure of proximity. We used the agglomeration schedule (i.e., the change in agglomeration coefficients as the number of clusters increase) to determine the optimum number of clusters. Afterwards, each HD mutation gene carrier was assigned to one of the identified sub-groups by repeating the cluster analysis using the optimal number of clusters. Finally, we used Pearson's partial correlation analysis, including age, gender, site, and education as covariates of no interest, to examine how sub-group membership was correlated with behavioral performance and putamen volume as a marker of disease progression. Bonferroni correction was used to account for the number of correlation tests.

## Results

### Behavioral data

Fifteen participants scored low in performance (<50% accuracy) or performed a wrong condition and were thus excluded from the subsequent analysis (Table S1). It was furthermore examined whether this exclusion was dictated by group affiliation, which was not the case [chi^2^(2, *N* = 200) = 1.28, *p* = 0.53].

Descriptive information of the motor performance is provided in the Table S2. Between-group differences were assessed using factorial ANCOVA analysis. Significant performance differences were found among the groups only for the speed conditions [*F*_(2, 148)_ = 4.19, *p* = 0.017], as measured by the standard deviation of timing inaccuracy (i.e., the time between a button press and closest click). No between-group differences were observed for complexity [*F*_(2, 148)_ = 1.691, *p* = 0.181] or for the interaction between complexity and speed [*F*_(2, 148)_ = 1.734, *p* = 0.180]. To further investigate the significant between-group effects in speed, *post-hoc t*-tests (Bonferroni-corrected) were conducted for each pair of groups separately. Between-group differences in timing inaccuracy (SD) were observed for the two slow speed conditions (simple slow and complex slow) and only between HC and earlyHD (*p* < 0.05, Bonferroni-corrected). No significant differences were found between HC vs. preHD and between preHD vs. earlyHD. Groups did not differ in accuracy (i.e., percentage of correct responses), neither for the main effect of complexity, nor for the main effect of speed (both with *p* > 0.1).

Similarly to our previous study (Klöppel et al., 2009), a positive correlation was found in HD gene carriers between CPO and performance in both complex conditions (complex slow: *r* = 0.242, *n* = 78, *p* = 0.047, and complex fast: *r* = 0.289, *n* = 78, *p* = 0.017) as well as during simple slow (*r* = 0.252, *n* = 78, *p* = 0.038), as measured by the absolute values of the timing inaccuracies (SD). This suggests that HD gene carriers performed worse (i.e., became less accurate during tapping) with disease progression.

### GLM results

**Figure 2:**
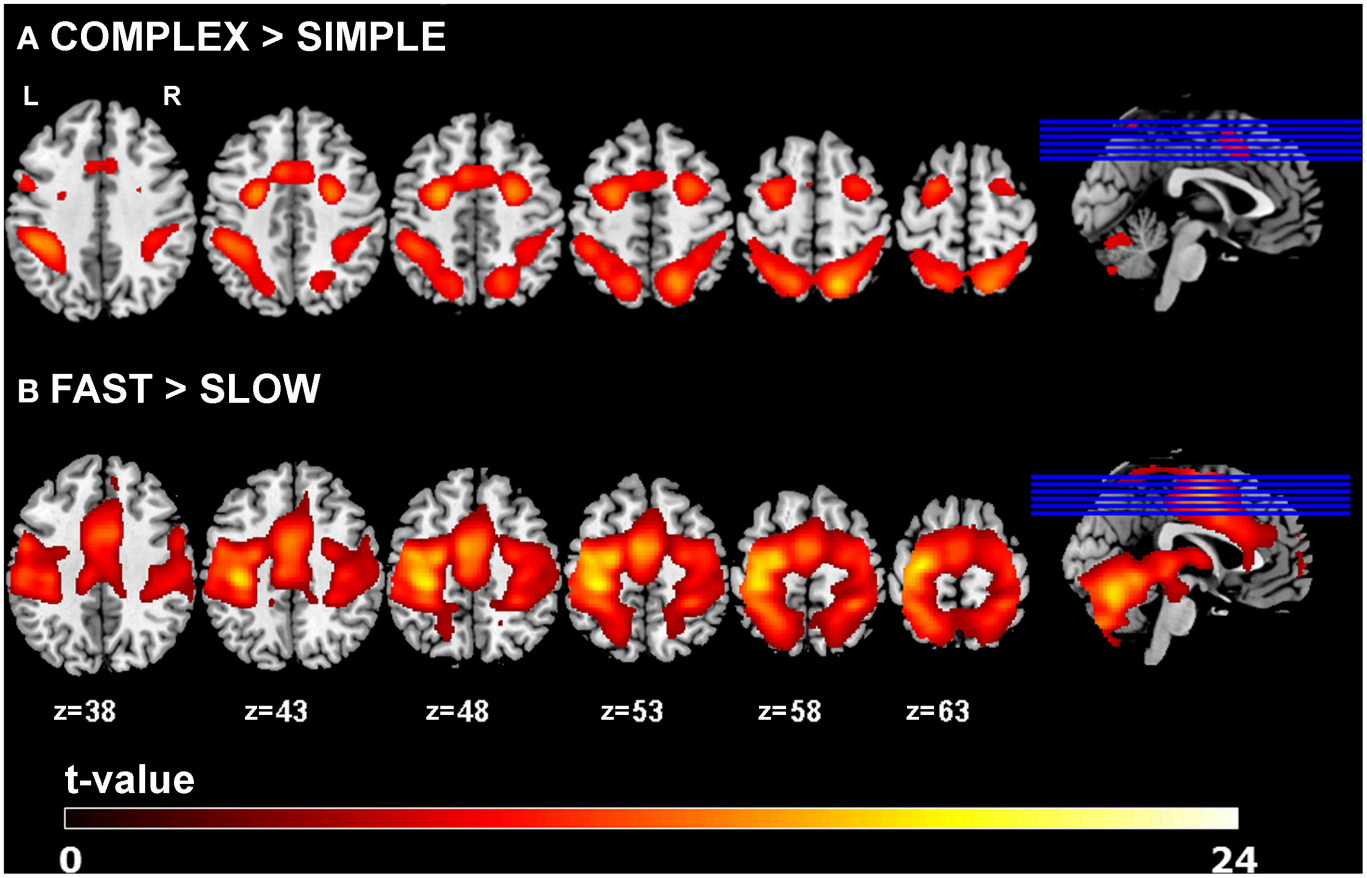
**GLM results**. Main effects of task for **(A)** complexity and **(B)** speed across all participants (*p* < 0.05 FWE-corrected, minimum cluster size *k* = 100).

Main effects of experimental task resulted in activations of left primary motor cortex (lM1), left pre-SMA (pSMA), left caudal SMA (cSMA), bilateral dorsal premotor cortex (lPMd, rPMd) and bilateral superior parietal cortex (lSPC, rSPC), which is in agreement with previous findings (Klöppel et al., 2009). Increased complexity of sequential movements resulted in stronger activations in the pSMA, bilateral PMd, and bilateral SPC, while increased speed of sequential movements led to stronger activation in cSMA and lM1 areas. Activation results are shown in Figure 2 and the corresponding regions of interest and their coordinates can be found in Table 2. No significant main effects of group or interactions between group and the two task conditions (i.e., complexity and speed) were found at *p* < 0.05 (FWE-corrected) using the GLM Flex tool.

### DCM results

**Figure 3:**
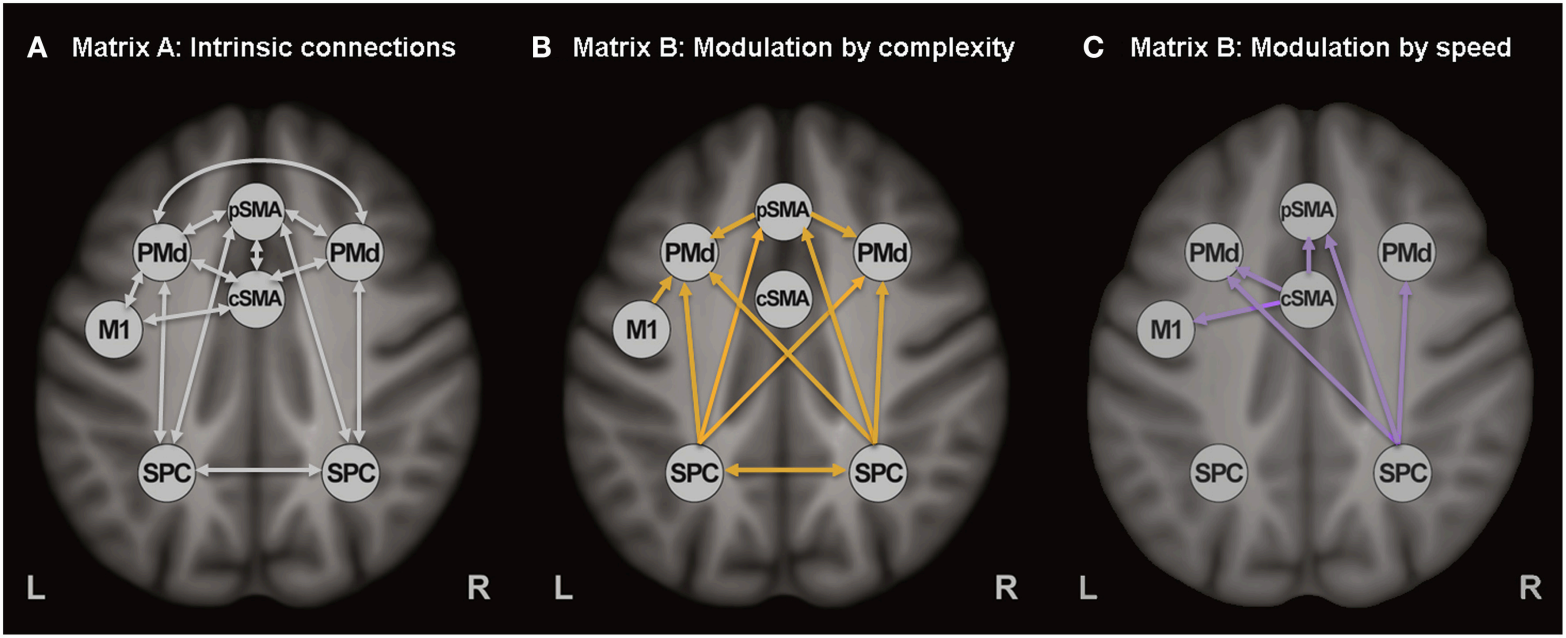
**Winning DCM model after ***post-hoc*** Bayesian model selection**. **(A)** Task-independent, intrinsic connections, **(B)** Modulatory connections (complexity), and **(C)** Modulatory connections (speed).

The diagnostics of each participant's DCM with regard to variance explained by the model and parameter estimability led to the exclusion of 35 participants (17 HC, 14 preHD, and 4 earlyHD). *Post-hoc* analysis revealed the same winning model across the three groups with the highest probability of (almost) 1. In the winning model (Figure 3), only a small number of modulatory connections were removed, such as the connections from the rPMd cortex toward the other regions, as well as the modulatory effects of complexity on the neural coupling from pSMA toward both bilateral SPC and cSMA. In an exploratory manner, the *post-hoc* optimization procedure was repeated for controls and HD gene carriers separately to ensure that the same winning model was identified for the patient group, which was the case. The posterior probabilities resulting from the *post-hoc* optimization across all subjects were further examined in quantitative terms using frequentist inference. Descriptive statistics of all intrinsic and modulatory parameters can be found in the (Table S3).

**Figure 4:**
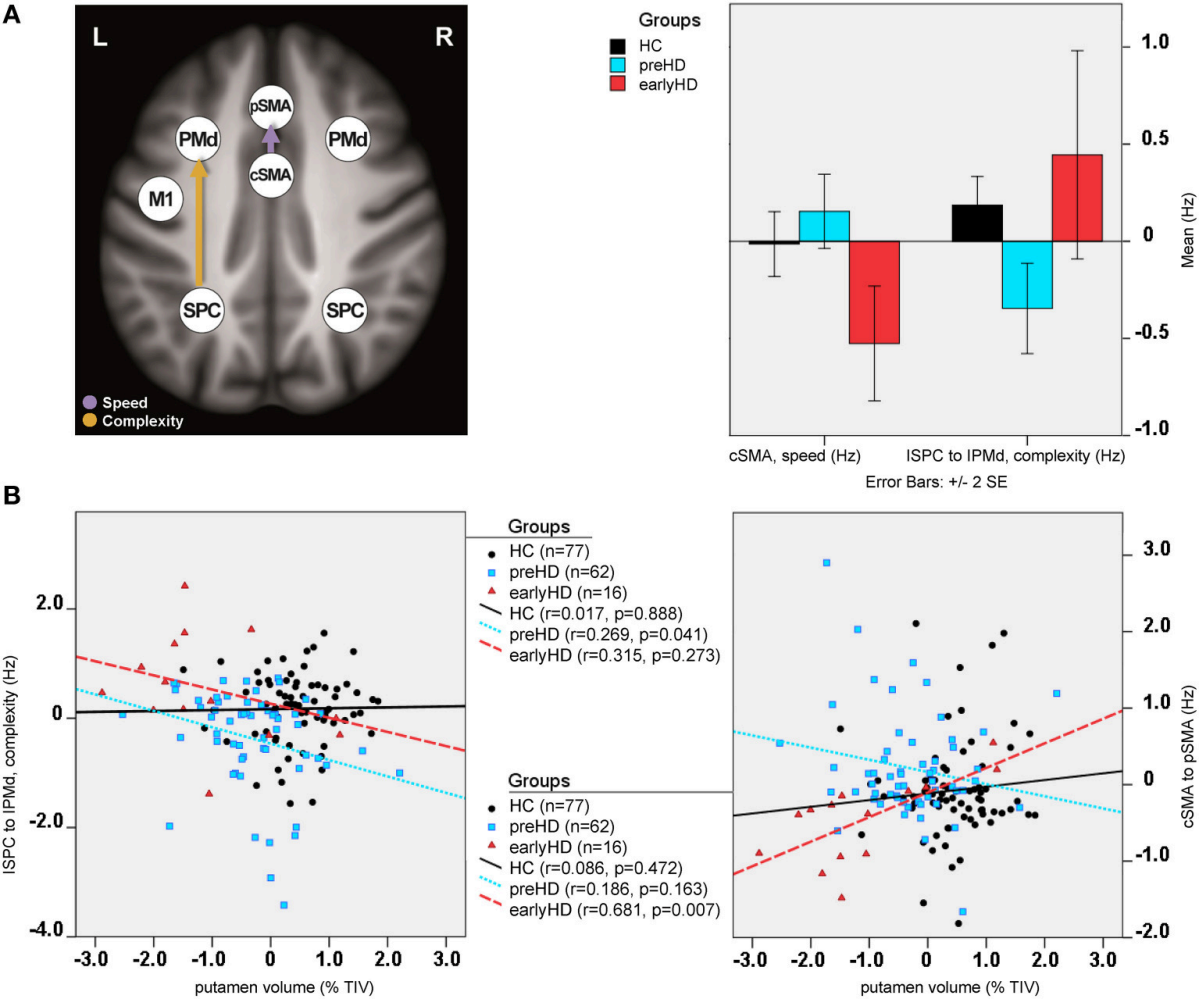
**DCM results: between-group differences**. **(A)** Differential modulatory effects driven by complexity and speed. **(B)** Correlation analysis.

Differences in effective connectivity between HC and HD gene mutation carriers were found only for the task-dependent, modulatory neural coupling from lSPC toward lPMd during complex conditions [*F*_(2, 148)_ = 4.43, *p* < 0.001; Figure 4A], but not for the endogenous connectivity (i.e., coupling that is constant across all experimental conditions). Specifically, *post-hoc* Bonferroni tests showed that effects of complexity from lSPC toward lPMd were inhibitory in preHD, which is in line with previous findings (Scheller et al., 2013), but became excitatory in earlyHD (*p* < 0.001, Bonferroni-corrected). Interestingly, a negative correlation was also found in all mutation carriers between lSPC-lPMd coupling and TIV-adjusted putamen volume (*r* = −0.302, *n* = 78, *p* = 0.007), suggesting that complex conditions led to increasingly excitatory neural coupling associated with decreasing putamen volume (Figure 4B). However, this effect was not correlated with any of the behavioral data. Still, this could partly be explained by the fact that our complex condition comprised a 4-item sequence (i.e., 4-2-3-1, see Figure S1), which might have not been too cognitively demanding.

Furthermore, modulatory effects during speed conditions significantly differed among the groups for the connections from rSPC to pSMA [*F*_(2, 148)_ = 4.10, *p* = 0.001], as well as from cSMA to pSMA [*F*_(2, 148)_ = 2.58, *p* = 0.021]. *Post-hoc* Bonferroni tests showed that these modulatory effects were expressed by excitatory rSPC to pSMA coupling in earlyHD as opposed to inhibitory in preHD and HC (*p* = 0.001), which, however, was not correlated to either putamen volume or behavioral performance. A trend of increased inhibitory coupling from cSMA toward pSMA, modulated by speed, was observed in earlyHD, relative to preHD and HC (*p* = 0.002), but this effect did not survive Bonferroni correction after accounting for the number of tests. Of note, the stronger inhibitory cSMA to pSMA connections were associated with decreased putamen volume (*r* = 0.632, *n* = 16, *p* = 0.032) and worse behavioral performance during both fast conditions (simple fast: *r* = −0.522, *n* = 16, *p* = 0.045, and complex fast: *r* = −0.724, *n* = 16, *p* = 0.018) in earlyHD, but not in preHD (*n* = 62; putamen: *r* = −0.186, *p* = 0.163, simple fast: *r* = −0.021, *p* = 0.873, and complex fast: *r* = −0.167, *p* = 0.211).

### Cluster analysis results

Three potentially meaningful clusters were identified, which were used to classify the HD mutation gene carriers accordingly (a scree plot of the agglomeration schedule is provided in the Figure S2). The group distribution was as follows: 23 participants were included in the first cluster, 46 in the second one, and 9 participants in the third cluster. The corresponding demographic, clinical, and motor performance information is provided in the Table S4. Sub-groups differed neither in their demographic (age, gender, and education) and clinical (DBS, CPO, and putamen volume) data, nor in their performance during scanning (i.e., means and SD of cue-response timing inaccuracies during the four movement conditions; Table S4).

**Figure 5:**
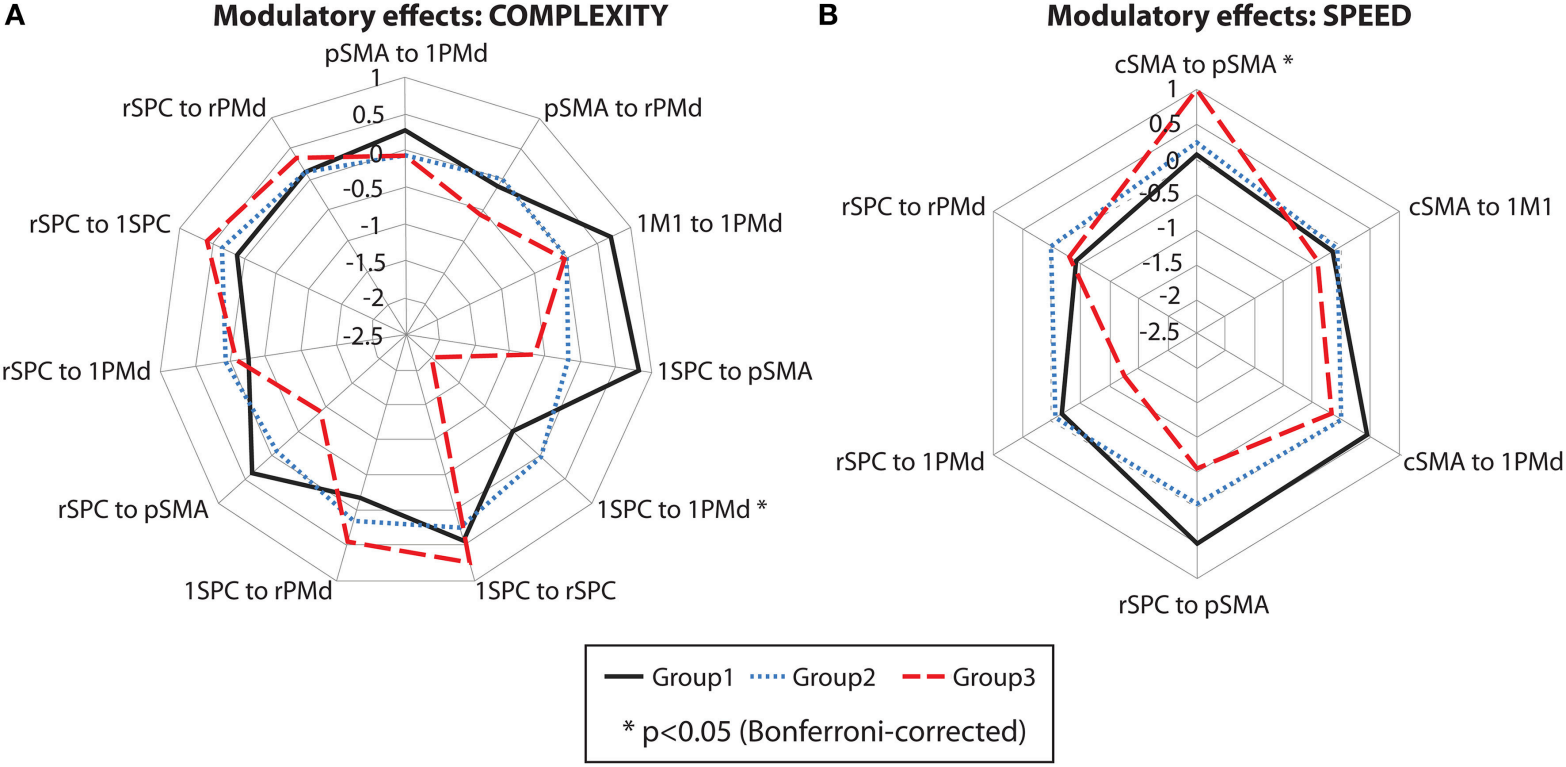
**Cluster analysis: connectivity profiles**. Modulatory effects of **(A)** complexity and **(B)** speed on neural coupling strengths in all cluster sub-groups. Significant effects are marked with an asterisk (*p* < 0.05, Bonferroni-corrected).

**Figure 6:**
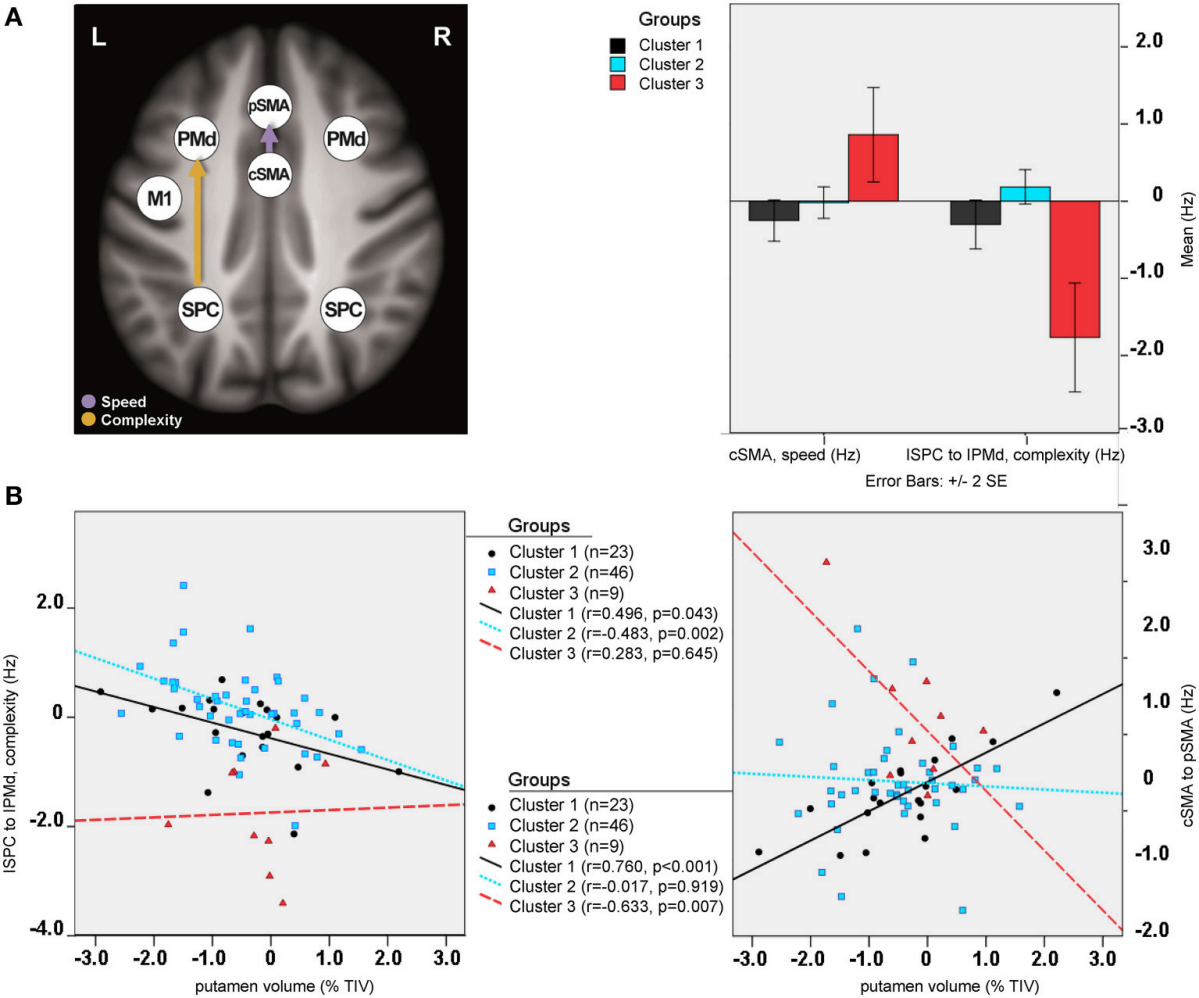
**Cluster analysis: sub-group differences**. **(A)** Differential modulatory effects driven by complexity and speed. **(B)** Correlation analysis.

Between-group differences were identified only for modulatory neural couplings (cSMA-pSMA modulated by speed: *F*_(2, 69)_ = 3.70, *p* = 0.003, and lSPC-lPMd modulated by complexity: *F*_(2, 69)_ = 8.99, *p* < 0.001), using ANCOVA analyses after adjusting for effects of age, gender, site, and education. Connectivity profiles for all modulatory connections are shown in Figure 5. Bonferroni *post-hoc* analyses revealed that group differences were present only between sub-group 3 (*N* = 9) and the other two sub-groups (Figure 6A). Specifically, there was a stronger excitatory coupling from cSMA toward pSMA modulated by speed and stronger inhibitory coupling from lSPC toward lPMd modulated by complexity in sub-group 3, relative to the other two sub-groups (all effects significant at *p* < 0.001). Interestingly, stronger excitatory coupling from cSMA toward pSMA was associated with decreased putamen volume (*r* = −0.633, *n* = 9, *p* = 0.007), as indicated by partial correlation analysis, adjusting for age, gender, education, and site (Figure 6B). With regard to the lSPC-lPMd connection, higher excitatory coupling in sub-groups 1 (*r* = −0.496, *n* = 23, *p* = 0.043) and 2 (*r* = −0.483, *n* = 46, *p* = 0.002), but not in sub-group 3 (*r* = 0.283, *n* = 9, *p* = 0.645), was associated with decreased putamen volume.

## Discussion

In this study, we sought to gain further insights into the neural circuitry of the motor network in Huntington's disease. For this purpose, a sequential finger-tapping fMRI task and DCM were used to assess the causal interactions among regions involved in both executive (movement speed) and cognitive (movement complexity) aspects of motor control. In the fMRI analysis, the same task-specific motor network was found as identified in our previous study (Klöppel et al., 2009). This included activations in pSMA, bilateral PMd, and bilateral SPC during complex tapping conditions, while the cSMA and lM1 were more strongly activated during fast finger tapping. Furthermore, it was shown here that, although preHD and earlyHD did not differ from each other in their behavioral performance, lower accuracy during tapping across all gene mutation carriers was associated with disease progression (i.e., cumulative probability of clinical onset). In the DCM analysis, on the other hand, the main focus was on the characterization of abnormal connectivity in the identified network of regions, which was common for both HD mutation gene carriers and healthy controls.

### Effective connectivity in HD

Our first aim was to extend on previously published DCM data in preclinical HD, which suggested the crucial role of premotor (i.e., PMd) and parietal areas (i.e., SPC), as part of fronto-parietal circuits, for the maintenance of motor functioning (Scheller et al., 2013). Our findings did not provide any evidence for the previously proposed compensatory role of premotor areas in preHD, characterized by an increased neural coupling from dorsal premotor cortex toward superior parietal cortex, which was regarded as indicative of neural reserve mechanisms that occurred during complex movements (i.e., high cognitive demand) in preHD individuals closer to clinical onset. However, it should be emphasized that the cognitive aspect in our experiment was less complex and might have been insufficiently demanding (i.e., participants in our study had to learn a complex sequence of 4 digits as compared to the 10-item sequence used in the previous study). Of note, a stronger inhibitory modulatory coupling was found from lSPC toward lPMd in preHD, relative to HC, which is in line with our previous findings (Scheller et al., 2013), but, interestingly, the reversed excitatory effect was also present in earlyHD. Furthermore, stronger excitatory effects from lSPC toward lPMd were associated with lower putamen volume in all gene carriers, which is only partly explained by group membership, as indicated by the substantially overlapping values for putamen volume between preHD and earlyHD (Figure 3B). Putamen volume was used as a disease marker, since striatal atrophy is a well-attested clinical hallmark of HD. Also, previous DTI data have confirmed that the putamen is interconnected with our regions of interest, including (but not limited to) the primary motor and premotor cortices and the supplementary motor area (Leh et al., 2007).

The present analysis also revealed that task-induced changes during speed conditions resulted in a stronger inhibitory coupling from cSMA to pSMA in those earlyHD patients who had lower putamen volume and performed worse at the fast motor conditions. However, this effect should be considered with caution because it reached only trend significance after correction for multiple comparisons. Also, the earlyHD group was overall slightly smaller in size than the healthy controls and the preHD, which might have introduced an additional bias. Nevertheless, we believe that the current study provides results that are complementary to our previous findings and suggests that the choice of experimental manipulation is critical for assessing and understanding the complex functional connectivity pattern between core regions maintaining motor function. It also points to the heterogeneity inherent across the HD gene mutation carriers and further supports the notion that identifying sub-groups of patients that are not merely defined according to clinical onset would be beneficial for future interventions.

### Cluster analysis for HD sub-group classification

The second aim of our study was to explore the application of a hierarchical cluster analysis based on the DCM intrinsic and task-specific parameters in an attempt to identify clinically meaningful sub-groups within the HD gene carrier group. Cluster analysis approaches based on structural imaging data have already proven useful for stratification of patient populations and predictions of clinical outcomes in the context of aging and Alzheimer's disease (Nettiksimmons et al., 2010; Damian et al., 2013; Peter et al., 2014; Quaranta et al., 2014). However, to our knowledge, this is the first study using task-based DCM neural couplings for classification of clinical sub-groups.

Hierarchical clustering is an unsupervised clustering approach, which may render the selection of optimal number of clusters arbitrary, as it is highly dependent on the similarity measures used. Here, the HD gene carriers were divided into three different clusters after the visual inspection of the dendrogram and considering the change in agglomeration coefficients as the number of clusters increased. This is also consistent with previous divisions of pre-symptomatic HD into preHD-A (further from predicted diagnosis) and preHD-B (nearer) and of manifest HD into stage 1 (HD1) and stage 2 (HD2), depending on their total functioning capacity scores (Tabrizi et al., 2009). Of note, only early stage HD individuals were included in the current analysis, who were initially recruited as preHD but have converted during the course of the TrackOn-HD study. After close inspection of the clusters, it is of note that DCM-based cluster membership was not merely explained by disease burden and did not overlap with the differentiation between preHD and earlyHD.

The differences in DCM parameters among the three sub-groups reflected the same variation in modulatory neural coupling as observed in the DCM-based ANCOVA analysis, using the initially defined membership (i.e., HC, preHD, and earlyHD). It should be noted that the cluster consisting of 9 individuals (7 preHD-B and 2 preHD-A) differed significantly from the other two clusters (*N* = 23 and *N* = 46), but at the same time showed the opposite effects than those observed in earlyHD. Specifically, neural coupling strengths from left parietal regions toward premotor areas, modulated by complex tapping movements, was excitatory in nature in earlyHD and inhibitory in cluster sub-group 3. On the other hand, fast tapping movements differentially modulated the neural coupling from cSMA to pSMA in such a way that it was inhibitory in earlyHD, relative to preHD and HC, and excitatory in sub-group 3, relative to the other two clusters.

Clearly, this provides further support for the heterogeneity in neural circuits across the HD disease spectrum but, due to the lack of clear correlations with behavioral measures of speed and complexity, does not provide firm evidence for compensatory mechanisms. Nevertheless, the excitatory neural coupling from lSPC to lPMd, which increased with lower putamen volume, together with the association of CPO with lower behavioral performance, may possibly point to an attempted (as opposed to successful) compensatory mechanism (for an in-depth discussion of successful vs. attempted compensation please refer to Scheller et al., 2014). Of note, some regions involved in motor control, such as the cSMA and rSPC (Klöppel et al., 2009), as well as the bilateral PMd (Scheller et al., 2013), seem to be essential for maintaining motor functioning, and increased cortical recruitment has also been observed in anterior cingulate-frontal-motor-parietal cortex in HD during a working memory task (Georgiou-Karistianis et al., 2007). In a resting-state fMRI study in HD (Werner et al., 2014), however, increased functional connectivity in motor and parietal cortices was associated with motor impairments, pointing that cortical over-recruitment may not necessarily reflect compensation but could also be indicative of a dysfunction due to HD disease-related deficits. Thus, compensation could also be characterized by down-regulation or disengagement of brain regions (Cox et al., 2015). Alternatively, increased cortical activations may be beneficial in some individuals but become insufficient for retaining high level of functioning in others, as disease progresses.

### Limitations and future directions

Altogether, the present study showed that DCM could successfully be applied to assess aberrant effective connectivity in Huntington's disease. Based on directed neural coupling strengths and their change caused by experimental perturbations, a potentially useful classification of HD mutation gene carriers was identified. However, certain limitations need to be mentioned in this regard. Clusters were defined in an exploratory manner and while an interesting pattern of DCM-based classification was observed, the clinical value of our findings still needs to be evaluated. It is still to be investigated whether cluster membership remains stable over time and whether it is predictive of future clinical outcomes (e.g., conversion to HD, disease progression, and domain-specific changes reflected by behavioral markers). Future studies focusing on longitudinal data should address these issues and should also aim at providing more mechanistic, biologically-relevant insights into the neural circuitry in HD, differentiating between maladaptive vs. compensatory mechanisms, which will be of great importance for future targeted interventions.

### Conflict of interest statement

The authors declare that the research was conducted in the absence of any commercial or financial relationships that could be construed as a potential conflict of interest.

## Supplementary material

Figure S1 **Experimental design: sequential motor finger-tapping task**.

Figure S2 **Scree plot: change in agglomeration coefficients (y-axis) as the number of clusters increase (x-axis)**.

Table S1 **Exclusion criteria**.

Table S2 **Descriptive information about motor performance**.

Table S3 **Descriptive statistics of DCM connection strengths**.

Table S4 **Cluster analysis: demographic, clinical, and motor performance information (HD gene mutation carriers only)**.
