# Supplementary figures and images for: Detection of Motor Changes in Huntington's Disease Using Dynamic Causal Modeling
Source: Front Hum Neurosci. 2015 Nov 25;9:634. doi: 10.3389/fnhum.2015.00634 (PMC4658414; doi:10.3389/fnhum.2015.00634)

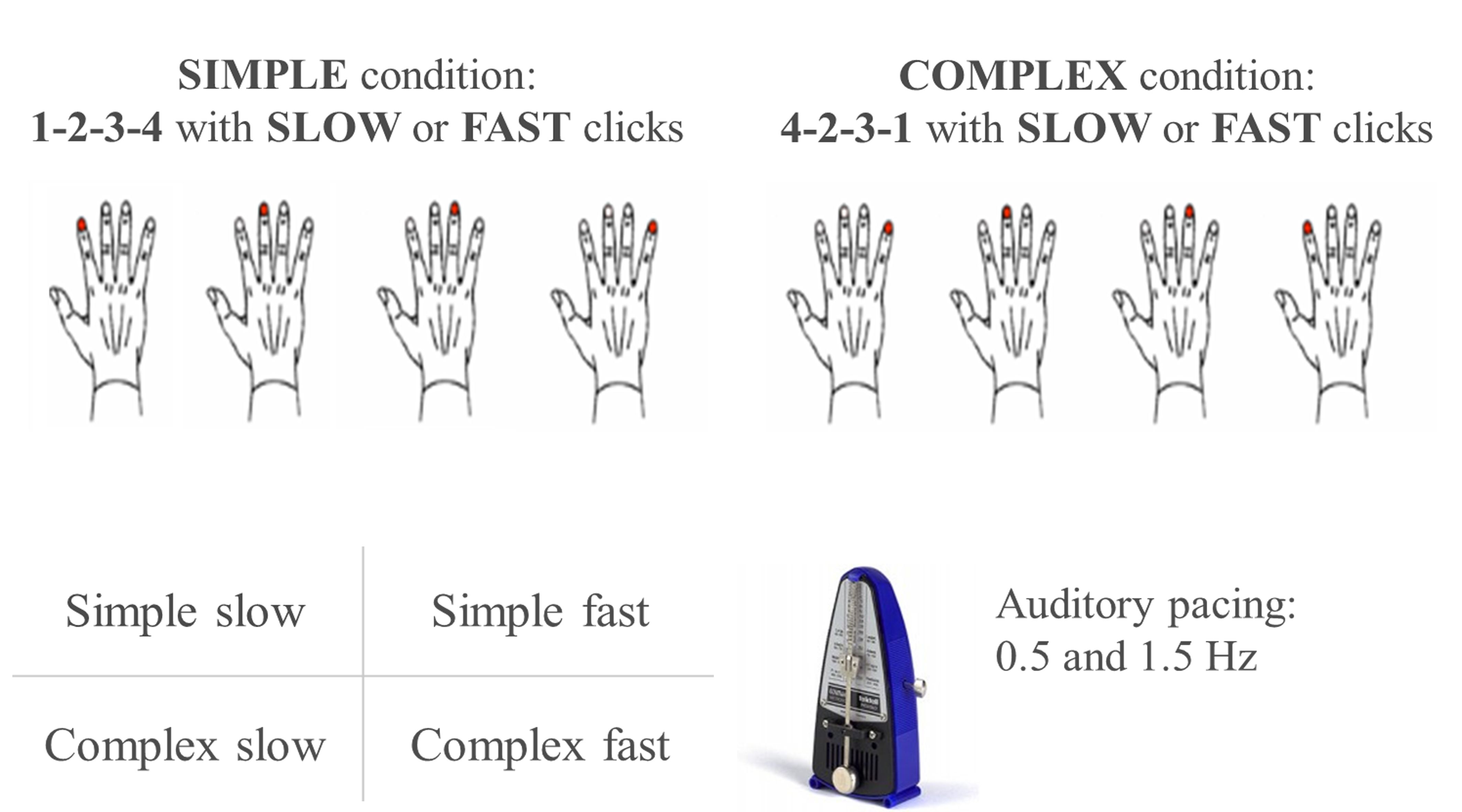

Supplement: Figure S1 — Experimental design: sequential motor finger-tapping task. [file Image1.TIF]

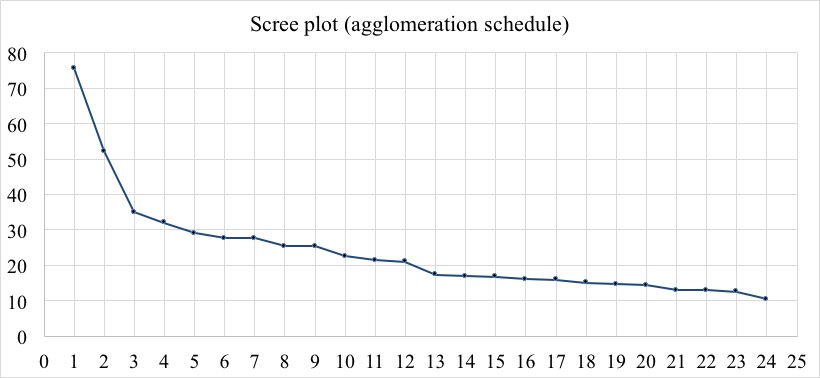

Supplement: Figure S2 — Scree plot: change in agglomeration coefficients (y-axis) as the number of clusters increase (x-axis). [file Image2.JPEG]
