# Supplementary material for: Detection of Motor Changes in Huntington's Disease Using Dynamic Causal Modeling
Source: Front Hum Neurosci. 2015 Nov 25;9:634. doi: 10.3389/fnhum.2015.00634 (PMC4658414; doi:10.3389/fnhum.2015.00634)
Supplement: Table S1 — Exclusion criteria. [file Table1.DOC]

# Supplementary Material

| **Table S1**. Exclusion criteria   | **Reasons for exclusions** | **HC** | **preHD** | **earlyHD** | | --- | --- | --- | --- | | Left-handedness | 7 | 5 | 4 | | Technical issues, image artifacts, medication | 2 | 4 | 5 | | Missing or corrupt data | 3 | 3 | 3 | | Poor behavioral performance | 8 | 4 | 3 | | DCM quality check failed | 17 | 14 | 4 | | **Total number of exclusions (86 out of 241)** | 37 | 30 | 19 | | **Final sample included in the study (N=155)** | 77 | 62 | 16 | |
| --- | --- | --- | --- | --- | --- | --- | --- | --- | --- | --- | --- | --- | --- | --- | --- | --- | --- | --- | --- | --- | --- | --- | --- | --- | --- | --- | --- | --- | --- | --- | --- | --- |
