# Supplementary material for: Detection of Motor Changes in Huntington's Disease Using Dynamic Causal Modeling
Source: Front Hum Neurosci. 2015 Nov 25;9:634. doi: 10.3389/fnhum.2015.00634 (PMC4658414; doi:10.3389/fnhum.2015.00634)
Supplement: Table S2 — Descriptive information about motor performance. [file Table2.DOC]

# Supplementary Material

**Table S2**. Descriptive information about motor performance

|  | **HC (n=77)** | **preHD (n=62)** | **earlyHD (n=16)** |
| --- | --- | --- | --- |
| **Cue-response intervals (ms), SD, simple slow** | 133 ± 44 (55:252) | 137 ± 40 (45:248) | 164 ± 54 (34:200) |
| **Cue-response intervals (ms), SD, simple fast** | 82 ± 40 (34:200) | 88 ± 39 (29:205) | 85 ± 24 (34:200) |
| **Cue-response intervals (ms), SD, complex slow** | 144 ± 56 (47:222) | 156 ± 42 (77:299) | 184 ± 56 (85:260) |
| **Cue-response intervals (ms), SD, complex fast** | 89 ± 42 (27:203) | 94 ± 35 (30:179) | 102 ± 27 (42:137) |
| **Correct responses (%), simple slow** | 98 ± 4 (78:100) | 98 ± 4 (80:100) | 98 ± 3 (89:100) |
| **Correct responses (%), simple fast** | 96 ± 7 (72:100) | 98 ± 5 (77:100) | 98 ± 4 (84:100) |
| **Correct responses (%), complex slow** | 98 ± 4 (80:100) | 98 ± 3 (80:100) | 98 ± 4 (92:100) |
| **Correct responses (%), complex fast** | 95 ± 6 (75:100) | 95 ± 6 (74:100) | 87 ± 6 (74:100) |

Values are given in means ± SD (range). Abbreviations: HC = healthy controls; preHD = pre-symptomatic HD; earlyHD = early manifest HD
