# Supplementary material for: Detection of Motor Changes in Huntington's Disease Using Dynamic Causal Modeling
Source: Front Hum Neurosci. 2015 Nov 25;9:634. doi: 10.3389/fnhum.2015.00634 (PMC4658414; doi:10.3389/fnhum.2015.00634)
Supplement: Table S3 — Descriptive statistics of DCM connection strengths. [file Table3.DOC]

# Supplementary Material

# Table S3. Descriptive statistics of DCM connection strengths

|  | **HC (N=77)** | | **preHD (N=62)** | | **earlyHD (N=16)** | |
| --- | --- | --- | --- | --- | --- | --- |
|  | **Mean** | **SD** | **Mean** | **SD** | **Mean** | **SD** |
| **Intrinsic connections (A-Matrix)** | | | | | | |
| pSMA to cSMA | -.069* | .144 | -.064* | .149 | -.104 | .129 |
| pSMA to lPMd | .031 | .158 | .069* | .131 | .099 | .161 |
| pSMA to lSPC | .148* | .197 | .193* | .180 | .118* | .100 |
| pSMA to rSPC | .195* | .189 | .124* | .196 | .159* | .091 |
| pSMA to rPMd | .060 | .162 | .060 | .157 | -.037 | .118 |
| cSMA to pSMA | .208* | .223 | .215* | .209 | .156 | .199 |
| cSMA to lM1 | .019 | .139 | .033 | .131 | .080 | .132 |
| cSMA to lPMd | .249* | .217 | .239* | .207 | .190* | .152 |
| cSMA to rPMd | .285* | .224 | .279* | .199 | .240* | .237 |
| lM1 to cSMA | .222* | .185 | .219* | .151 | .228* | .150 |
| lM1 to lPMd | .340* | .299 | .320* | .244 | .407* | .243 |
| lPMd to pSMA | .019 | .174 | .081* | .180 | .063 | .180 |
| lPMd to cSMA | -.076* | .141 | -.057* | .105 | -.093 | .140 |
| lPMd to lM1 | -.064* | .128 | -.057* | .116 | -.080 | .141 |
| lPMd to lSPC | .229* | .201 | .233* | .153 | .266* | .239 |
| lPMd to rSPC | .222* | .197 | .141* | .212 | .198* | .175 |
| lPMd to rPMd | .066 | .202 | .054 | .209 | .132 | .146 |
| lSPC to pSMA | -.105* | .284 | -.112 | .308 | -.104 | .239 |
| lSPC to lPMd | -.071 | .238 | -.121* | .225 | -.044 | .214 |
| lSPC to rSPC | .118* | .197 | .049 | .178 | .149 | .177 |
| lSPC to rPMd | .037 | .237 | .058 | .262 | -.055 | .251 |
| rSPC to pSMA | -.092 | .335 | -.095 | .297 | -.098 | .224 |
| rSPC to lPMd | -.068 | .317 | -.120* | .229 | -.051 | .247 |
| rSPC to lSPC | -.021 | .248 | .008 | .206 | .092 | .199 |
| rSPC to rPMd | .030 | .289 | .059 | .257 | -.020 | .259 |
| rPMd to pSMA | -.008 | .153 | -.003 | .174 | -.044 | .135 |
| rPMd to cSMA | -.066* | .121 | -.062* | .127 | -.082 | .108 |
| rPMd to lPMd | .007 | .174 | -.008 | .169 | .064 | .139 |
| rPMd to lSPC | .148* | .153 | .145* | .147 | .146 | .241 |
| rPMd to rSPC | .161* | .147 | .152* | .176 | .157* | .101 |
| **Modulatory connections (B-Matrix, complexity)** | | | | | | |
| pSMA to lPMd | -.039 | .699 | -.005 | .552 | .176 | .710 |
| pSMA to rPMd | .111 | .543 | -.132 | .624 | .087 | .658 |
| lM1 to lPMd | .269* | .625 | .152 | .707 | .439* | .872 |
| lSPC to pSMA | .067 | .654 | .041 | 1.085 | .064 | .618 |
| lSPC to lPMd | -.018 | .643 | -.544* | .937 | .311* | 1.070 |
| lSPC to rSPC | .321* | .552 | .318* | .643 | .469* | .705 |
| lSPC to rPMd | .184 | .716 | .178 | .955 | -.301 | .639 |
| rSPC to pSMA | .117 | .897 | .038 | .780 | -.362 | .711 |
| rSPC to lPMd | -.040 | .722 | -.067 | .700 | .052 | .504 |
| rSPC to lSPC | .328* | .659 | .393* | .575 | -.014 | .909 |
| rSPC to rPMd | -.038 | .714 | .237 | .679 | -.085 | .466 |
| **Modulatory connections (B-Matrix, speed)** | | | | | | |
| cSMA to pSMA | .266* | .723 | .446* | .784 | -.245* | .578 |
| cSMA to lM1 | -.192* | .500 | -.192* | .578 | .111 | .647 |
| cSMA to lPMd | .194 | .700 | .016 | .836 | .293 | .809 |
| rSPC to pSMA | -.241 | .695 | -.060 | .723 | .510* | .595 |
| rSPC to lPMd | -.325* | .662 | -.321* | .665 | .133 | .914 |
| rSPC to rPMd | .137 | .700 | -.156* | .884 | -.149* | .162 |

* Connections that significantly differed from zero (based on one-sample t-tests with a significance threshold of p<0.001 after Bonferroni correction) are marked with an asterisk.

Abbreviations: SD = standard deviation; HC = healthy controls; preHD = pre-symptomatic HD; earlyHD = early manifest HD; cSMA = causal supplementary motor area; pSMA = pre-SMA; r=right; l=left; PMd=dorsal premotor cortex; M1 = primary motor cortex; SPC = superior parietal cortex
