# Supplementary material for: Detection of Motor Changes in Huntington's Disease Using Dynamic Causal Modeling
Source: Front Hum Neurosci. 2015 Nov 25;9:634. doi: 10.3389/fnhum.2015.00634 (PMC4658414; doi:10.3389/fnhum.2015.00634)
Supplement: Table S4 — Cluster analysis: demographic, clinical, and motor performance information (HD gene mutation carriers only). [file Table4.DOC]

# Supplementary Material

**Table S4**. Cluster analysis: demographic, clinical and motor performance information (HD gene mutation carriers only)

|  | **Cluster 1 (n=23)** | **Cluster 2 (n=46)** | **Cluster 3 (n=9)** |
| --- | --- | --- | --- |
| **Age (years)** | 44.25 ± 9.61 (27:61) | 42.25 ± 8.62 (24:67) | 46.18 ± 6.87 (31:50) |
| **Gender (F/M)** | 6 / 17 | 26 / 20 | 4 / 5 |
| **CAG length** | 42.96 ± 1.85 (40:46) | 43.28 ± 2.68 (39:50) | 43.44 ± 2.30 (40:47) |
| **CPO** | 0.30 ± 0.20 (0.02:0.75) | 0.24 ± 0.18 (0.03:0.83) | 0.22 ± 0.10 (0.11:0.37) |
| **Disease burden score*** | 317 ± 52 (208:401) | 311 ± 64 (182:457) | 317 ± 49 (225:377) |
| **Putamen (TIV-adjusted)** | 0.49 ± 0.11 (0.24:0.75) | 0.48 ± 0.09 (0.29:0.67) | 0.52 ± 0.07 (0.36:0.62) |
| **Cue-response intervals (ms), SD, simple slow** | 159 ± 48 (72:240) | 137 ± 43 (45:257) | 129 ± 25 (97:178) |
| **Cue-response intervals (ms), SD, simple fast** | 86 ± 33 (29:171) | 89 ± 39 (49:205) | 77 ± 32 (53:159) |
| **Cue-response intervals (ms), SD, complex slow** | 170 ± 62 (77:299) | 163 ± 40 (95:260) | 147 ± 30 (94:180) |
| **Cue-response intervals (ms), SD, complex fast** | 97 ± 35 (30:166) | 97 ± 35 (55:179) | 86 ± 21 (51:119) |
| **Correct responses (%), simple slow** | 98 ± 3 (89:100) | 98 ± 4 (80:100) | 99 ± 3 (93:100) |
| **Correct responses (%), simple fast** | 98 ± 5 (80:100) | 98 ± 4 (80:100) | 95 ± 8 (77:100) |
| **Correct responses (%), complex slow** | 98 ± 5 (80:100) | 98 ± 3 (87:100) | 97 ± 3 (90:100) |
| **Correct responses (%), complex fast** | 89 ± 3 (74:100) | 94 ± 5 (80:100) | 97 ± 3 (93:100) |

*DBS = age x (CAG length - 35.5) (Penney et al., 1997). Values are given in means ± SD (range), where applicable.

Abbreviations: F = female; M = male; CAG = trinucleotide; CPO = cumulative probability of clinical onset; TIV = total intracranial volume
